# Symbol-value association and discrimination in the archerfish

**DOI:** 10.1371/journal.pone.0174044

**Published:** 2017-04-05

**Authors:** Naomi Karoubi, Tali Leibovich, Ronen Segev

**Affiliations:** 1 Life Sciences Department and Zlotowski Center for Neuroscience, Ben Gurion University of the Negev, Beer Sheva, Israel; 2 Psychology Department and the Brain and Mind Institute, The University of Western Ontario, London, Canada; Universita degli Studi di Padova, ITALY

## Abstract

One of the most important aspects of mathematical cognition in humans is the ability to symbolically represent magnitudes and quantities. In the last 20 years it has been shown that not only humans but also other primates, birds and dolphins can use symbolic representation of quantities. However, it remains unclear to what extent this ability is spread across the animal kingdom. Here, by training archerfish to associate variable amounts of rewards with different geometric shapes, we show for the first time that lower vertebrates can also associate a value with a symbol and make a decision that maximizes their food intake based on this information. In addition, the archerfish is able to understand up to four different quantities and organize them mentally in an ordinal manner, similar to observations in higher vertebrates. These findings point in the direction of the existence of an approximate magnitude system in fish.

## Introduction

From counting eggs [1] to picking the pool with the most fish [2], to fighting an intruder lion only if enough allies are present [3], all of these behaviors reveal an innate notion of quantities in animals from insects [4] to primates [5]. Furthermore, more complex numerical abilities such as ordinal judgment or the addition of two quantities have been extensively investigated in species like birds, bees and non-human primates [6–8] and were found in many cases to equal at least the abilities of human infants [9].

Fishes also demonstrate a natural ability to assess quantities. For example, it has been demonstrated that in various species such as mosquitofish or angelfish, an individual will spontaneously join the bigger of two shoals in an attempt to avoid predators, but only when the difference in the number of individuals is significant and enables an easy discrimination [10–12]. In addition, other fish can also discriminate small quantities of objects [13,14], recognize the door with the correct number of symbols [15] or encode ordinal information [16]. They are able to perform above chance even when not given access to all the items together [17] or when they are deprived of the sense of sight [13].

All the previous studies used relative numerosity judgments with non-symbolic stimuli (although see [16]). Eliminating the possible influence of continuous magnitudes in numerosity comparison tasks is very difficult. Efforts were made to negate this influence by cleverly manipulating the relationship between number and continuous magnitudes so that continuous magnitudes will not be a predictive cue of numerosity. However, it has been demonstrated that even under such conditions, continuous magnitudes might influence performance [18]]. This is true for our study as well, although our work is the first to use abstract symbols associated with quantities.

*Homo sapiens* has developed advanced mathematical abilities in a way not observed in other animals, and it started by simply associating a quantity with a symbol. This involves the capacity to assign a value to an abstract symbol and then manipulate these representations as though they were the quantities themselves. Being able to tell for example which of two Arabic numerals is larger is not innate but requires learning. Traditionally it was claimed that this ability emerged with language and thus could not develop in animals lacking language [19,20].

Recently, however, studies have shown that a few animals can represent numerosities in a symbolic manner [21–27]. Monkeys were shown to be able to choose between two alternatives and pick the one that provided the largest food reward [8,28,29]. Chimpanzees can add Arabic numerals to obtain a reward [5]. Pigeons can associate a number of seeds with a symbol and peck the one that leads to a larger reward [30], and a parrot was reported to have learned to associate the vocalization of numerals with the corresponding number of objects [31].

All these results have contributed to a better understanding of the evolution of mathematical cognition. However, it remains unclear how widespread the ability to associate quantities and symbolic representations is among vertebrates. In particular, we do not know whether evolutionary branches that diverged long ago from mammals and birds also possess this ability. This question is interesting since it may provide clues to the anatomical locations in the brain that support these cognitive capabilities. However one should keep in mind that it is also possible that numerical abilities may be the result of convergent evolution and that different clades developed different mechanisms to solve numerical problems.

To address this question, we used the unique capability of the archerfish (*Toxotes chatareus*) to shoot a jet of water at targets presented on a computer monitor. We trained the fish to associate a quantity of food pellets with symbols and choose the target that was associated with the highest value of reward.

## Methods

### Animals

All the experiments were approved by the Ben-Gurion University of the Negev Animal Care and Use Committee and were in accordance with the laws of the State of Israel. A total of twelve adult archerfish, *Toxotes chatareus*, 6 to 12 cm, 10 to 15 g, were used for this study. They were purchased from a local animal seller. The fish were kept individually in water tanks (30 cm X 50 cm X 40 cm) filled with brackish water (2–2.5 g of a red sea salts mix per 1 liter of water) at 25°–28°C. The room was illuminated with artificial light on a 16/8 h day/night cycle. The water was filtered and oxygenated by an air pump.

### Training

**Fig 1:**
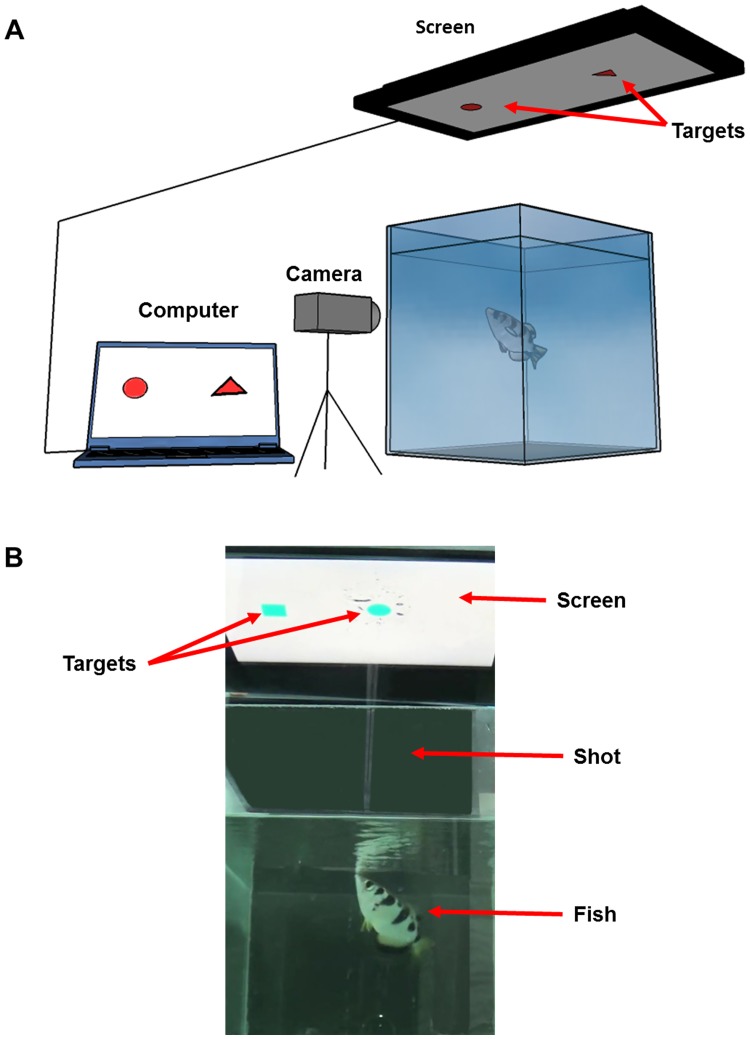
Experimental setup. **A.** Schematic representation of the experimental setup and the recording system. **B.** Photo of a real experiment (the black background was made darker by image manipulation to show the shot better).

The fish were trained to spit at targets presented on a LCD monitor (VW2245-T, 21.5", BenQ, Taiwan) placed 45 cm above the water level on transparent tempered glass (Fig 1). The food consisted of pellets of similar size of about 3 mm long (Red Parot Granulat, Tropical, Poland) allowing for clear monitoring of the amount received on each trial. Food pellets were simultaneously dropped manually by the experimenter on the side closest to her, and freely derived in the water until the fish ate them. Depending on the experiment, the fish received between 30 and 100 (+/- 20%) food pellets. They got most of their food intake from the experiments and only received the equivalent of 10–20 food pellets during the weekend. From experience, we estimate that the fish reach satiety after eating 100 +/- 20 food pellet per day and would stop spitting when reaching that level. Sessions were usually a minimum of 48 hours apart to avoid overfeeding and to maintain the fish in a responsive state. They were not fed between sessions during the week.

Initially, the fish were trained to shoot water at images of insects on the screen until they understood the task and shot at the targets every time. Aims at targets were rewarded with one food pellet. Then the images were replaced by a single black shape (square, triangle or circle alternatively). Again, the fish were rewarded with one food pellet for any shot at a target. Stimuli were presented using PowerPoint presentations (Microsoft, Seattle, WA, USA). Not all the fish were able to perform the whole set of experiments but since it was not critical for our study that the same fish performed all the different experiments, it did not influence our work. Some of them (4) died of natural causes. Some fish were dismissed because they stopped spitting or they lost their ability to shoot straight (6). This phenomenon is well known in the archerfish research community. Once a fish was dismissed, it was put in the aquarium with the fish for other projects.

All the experiments were recorded with a HD video camera (Handycam, HDR-CX240, Sony, Japan) at 25 frames per second. The videos were then manually analyzed with VirtualDub to measure reaction times. Reaction time was defined as the time difference between the onset of the targets and the beginning of the shot of water, similarly to common practice in fish research [32,33]. Because we signal the start of an experiment with a blinking square, fish are already paying attention to the screen when the targets appear. Hence there is no “dead time” when the fish needs to notice the stimuli like in Mamuneas’ experiment [32] for example, and thus the reaction time can be a good measure of cognitive performance.

### Behavioral experiments

A session was conducted during a single day and consisted of 36 trials; the fish generally responded 70% to 100% of the trials. The appearance of the targets was preceded by a black square (5 cm in height) that blinked three times in the center of the screen, signaling the beginning of a new trial to the fish. The targets were left on the screen for a maximum of two minutes or until the fish shot at one target. The stimuli were shapes (square, circle and triangle) of the same height (2 cm), which were either green, red or black during the experiments, depending on the fish's preference (see below). The value associated with each target varied across experiments. The positions of the target changed randomly between trials and sessions to avoid bias toward specific locations. They were positioned far enough from each other (minimum 3 cm) to eliminate any doubt as to which target was selected. The experiment was conducted until the fish attained a steady state, defined as at least five days with success rate above 60%. Exception was made for the third experiment with quantities where most of the fish’s success rate stabilized around 50%.

Not all the fish were involved in every experiment with the different quantities but they always went through an extinction phase (see below) between experiments.

### Natural preference

**Fig 2:**
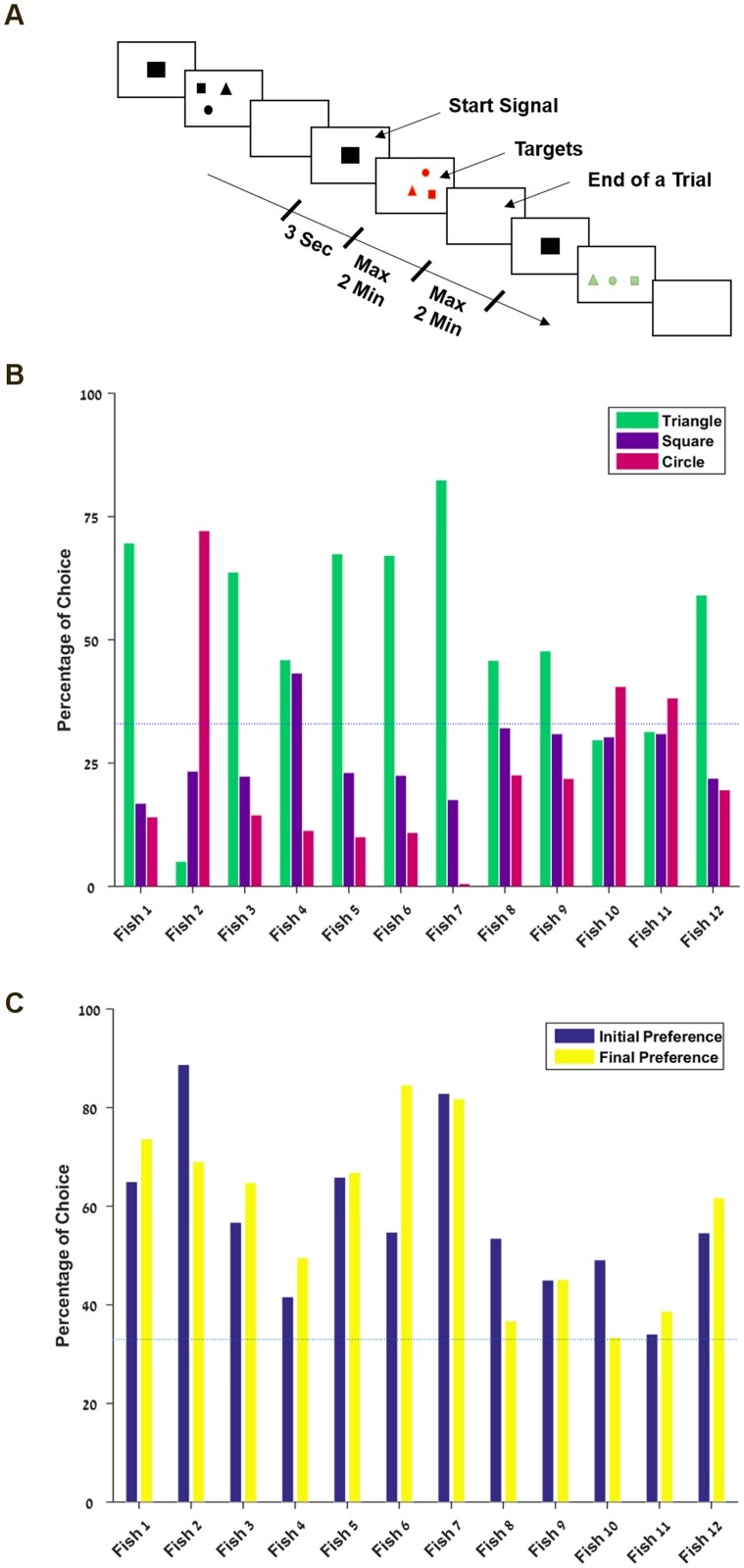
Fish show a variety of natural preferences. **A.** Example of sequence of stimuli presented during the determination of natural preference. **B.** Natural preference for shapes: percentage of choices for each target for the whole experiment. **C.** Stability of natural preference: average natural preference for the first and second half of the experiment. *The dashed line represents the chance level (33%)*.

In the first phase, we measured natural preferences for shapes and colors. These sessions consisted of 75 trials, where all three shapes—circle, square and triangle—were presented simultaneously during 15 seconds, at random positions to avoid bias for location. All the targets were the same color during one trial and the colors—green, red and black—were alternated every trial (Fig 2A), all colors appearing in equal proportions. Each shot at any target was rewarded with one food pellet, and the color and the shape chosen were recorded. This phase lasted in general from 5 to 10 days, and provided a dataset of 150 to 250 choices. All but three fish preferred triangles over squares over circles at a rate that greatly exceeded chance (Fig 2B). In addition, each fish had a preferred color (green, red or black) which was more or less evenly distributed among the fish. Fish 10 and 11, whose natural preferences were not as strong as the other fish, went through a short session (5 days) of preference reinforcement: the favorite target was rewarded with one food pellet and the least one with none. They reached a choice rate of 92% and 100% respectively.

### Persistence of natural preference

A comparison of the percentage of choices of the preferred target during the first and the second halves of the session showed that the natural preference remained strong over time although the fish received an equal reward for all targets (Fig 2C). We ran a two tails paired t-test to determine whether the initial and final choice rate were similar. We obtained a p-value of 0.7701, which indicates that the initial and final choice rate are indeed similar. These results confirm that the natural preference for shapes is persistent over time.

### Learning & value association

After the natural preference of each fish was determined, the experiments were conducted with its favorite color (green, black or red) and only two shapes (except for the last experiment), usually the circle and the triangle, depending on which one the fish preferred the most and the least. The positions of the targets followed the rules described earlier. In all these experiments, all the fish started with a clear preference for one target. It usually took a minimum of 6 sessions and up to 30 for them to learn to choose the other target.

To make sure that the fish could actually learn to choose their least favorite targets if it was rewarded, we first conducted a classical conditioning procedure. Here the least favorite target was rewarded with one food pellet whether the favorite one yielded no reward. During the experiments with two shapes, each target lead to a reward, the least favorite always being associated with the highest reward and the favorite with the lowest. The values of the rewards depended on the experiment. During the first experiment, we tested the ability for the fish to discriminate between 1 and 3 associated food pellets. In the second experiment, we tested it they could choose between 2 and 4, and in the latest, in order to check if they could discriminate when the difference between rewards was only one food pellet, we tested them with 3 and 4.

In the experiment with three shapes, the rewards were inversely proportional to the preference for the targets (1 for the triangle, 2 for the square and 3 for the circle). All the different combinations of targets were presented in equal proportion: the three pairs triangle-square, triangle-circle and square-circle, as well as the three shapes together.

### Extinction

In order to control that the fish were able to unlearn something they just learnt, and since we needed to be able to conduct several experiments with the same fish for this study and for future research, we subjected the fish to an extinction procedure between experiments [34]. During these sessions, intercalated between the experiments, the natural preference was reinforced again, in the opposite direction from the experiment they had just completed. This was obligatory since if we had simply switched the rewards associated with the targets, we would have ended up rewarding the favorite target with the highest amount of food. This congruency would have made it hard to determine whether the observed effect could be attributed to the magnitude of the reward or to the natural preference for the shape itself. In this phase, the fish were presented with the same targets but this time they were rewarded with one food pellet for their initially favorite target and zero for the other one, thus re-inducing their natural preference. This enabled us to run several experiments with the same fish by always associating the least preferred target with the highest amount of reward to avoid a congruence effect.

The summary of the participation of every fish to the different experiments is presented in Table 1. The fish always went through an extinction period between two experiments. The results of the many extinction procedures that were performed are not shown for the sake of clarity. The order of the experiments goes from left to right.

**Table 1:** Summary of fish participating in different experiments. Ext. stands for “extinction”. The + sign indicates that the fish took part in the experiment.

*Natural Preference*	*Learning and Extinction*	*1 versus 3*	*Ext*.	*2 versus 4*	*Ext*.	*3 versus 4*	*Ext*.	*1,2,3*
***Fish 1***	+	+	+	+	+			
***Fish 2***	+							
***Fish 3***	+	+	+	+	+			
***Fish 4***		+	+	+	+	+	+	+
***Fish 5***		+	+					
***Fish 6***		+	+					
***Fish 7***				+	+			
***Fish 8***				+	+	+	+	
***Fish 9***						+	+	
***Fish 10***						+	+	
***Fish 11***						+	+	+
***Fish 12***								***+***

### Statistical analysis

All statistical analyses were performed on Matlab. For the three experiments with two shapes, the chance level was 50%. For the experiment with three shapes, the chance level was set at 45.83%. This is because each session consisted of an equal proportion of each combination (1 versus 2, 1 versus 3, 2 versus 3, and the three shapes representing of 1, 2 and 3 altogether). The chance level for this experiment was thus the average of three time 50% and once 33%.

To determine the statistical significance of each experiment, we calculated the probability of obtaining the observed results by chance. Since each fish started with a strong preference for one target, we used its initial success rate—the choice rate during the first day- as the probability of success in the binomial distribution. We then simulated 10000 experiments of 36 shots—a number identical to the number of daily trials—and calculated the probability to observe a success rate equal or better than the one observed during the last day of the experiment. In this way, the p values were calculated as ‘number of simulated distributions with number of correct responses > = observed number of correct responses’. A p-value smaller than 0.005 indicates that we can reject the hypothesis that the observed initial and final results are identical.

## Results

### Learning and extinction

After establishing the fish’s natural preference for colors and shapes (see Method), the goal of this session was to test their ability to learn to spit at a target they did not choose spontaneously and then check if they were able do unlearn and go back to their initial preference.

**Fig 3:**
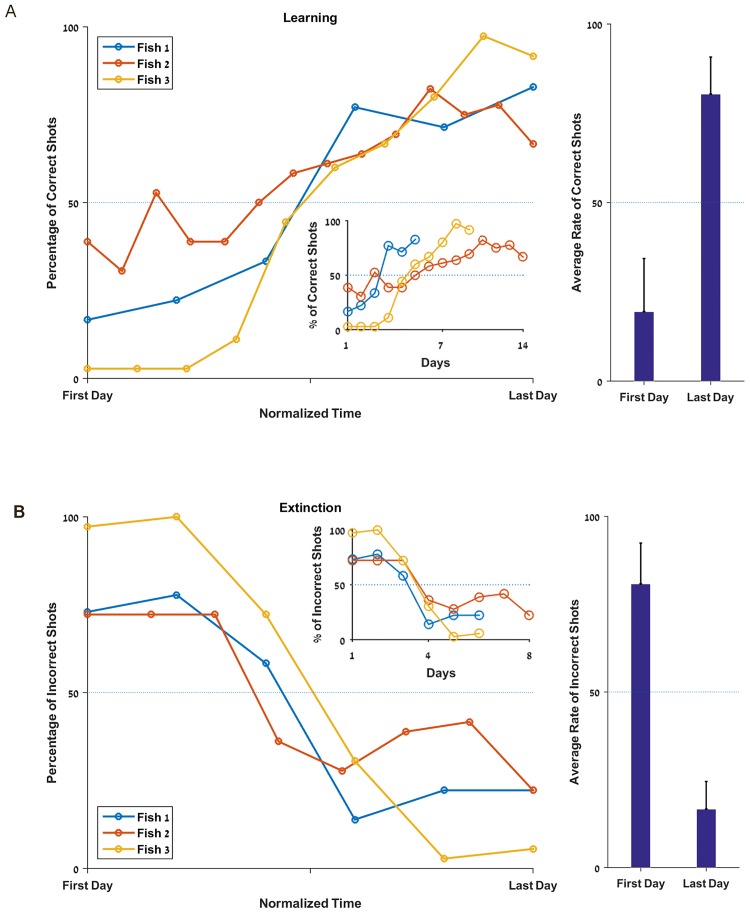
Fish are capable of learning and unlearning. **A.** Success rate for each fish during the learning experiment, with normalized axis for days; the inset shows the same results without normalization. **B.** Average rate of correct shots during learning on the first and last days. **C.** Failure rate for each fish during the extinction experiment, with normalized time; the inset shows the same results without normalization. **D.** Average rate of misses during extinction on the first and last days. *The dashed line represents the chance level (50%)*.

In the learning phase where only the least favorite target was rewarded, the fish started with a clear preference (Fig 3A) with at least 65% of the shots in the first session toward their preferred target. Nevertheless, all the fish learned to shoot at the rewarded target more than 70% of the time in less than 13 sessions, thus showing a clear ability to learn to choose the rewarded target (p<0.0001, Fig 3B).

Similarly, in the extinction phase, all the fish became proficient in five to seven sessions and went back to their natural preference (Fig 3C) with a minimum of a 70% choice of the favorite target (p<0.0001, Fig 3D).

#### Two shape-value association

**Fig 4:**
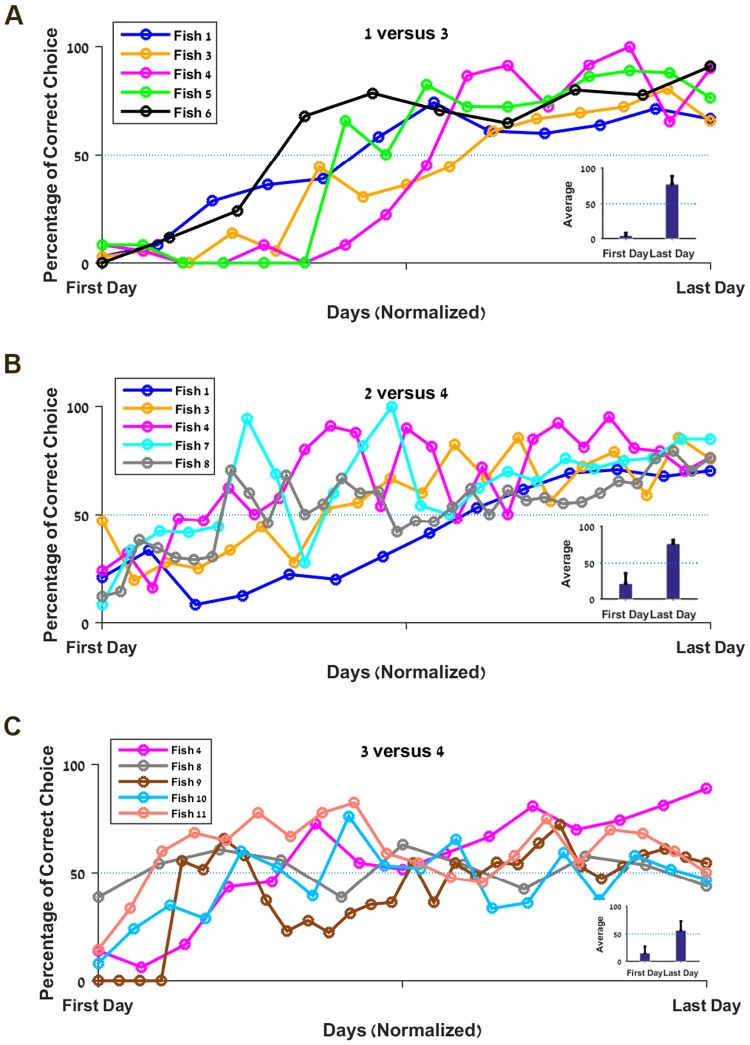
Fish can learn to associate value and shape. **A.** Success rate for each fish on the 1 versus 3 experiment over normalized time; inset: average success rate on the first and last day. **B.** Success rate for each fish on the 2 versus 4 experiment over normalized time; inset: average success rate on the first and last day. **C.** Success rate for each fish on the 3 versus 4 experiment over normalized time; inset: average success rate on the first and last day. *The dashed line represents the chance level (50%)*.

In these experiments with two targets, we tested whether the fish could associate different quantities of reward with each shape. In the first experiment, we tested if the fish would prefer three food pellets rather than one. Fig 4A shows that over the course of the experiment the fish changed their preference to shoot at the circle (rewarded with three) significantly more often than the triangle. The average choice rate on the last day was around 80%, which is significantly different from the initial 5% (p<0.0001, Fig 4A inset). Thus fish appeared to be able to distinguish between one and three food pellets, and change their shape preference accordingly.

After an extinction phase (see Methods), the fish had to choose between targets associated with two (triangle) and four food pellets (circle). Again, the results clearly showed that the fish were able to shift their preference (Fig 4B), since they preferred the circle at the end of the experiment (76% as compared to 12% initially, p<0.0001) with results significantly above chance (Fig 4B inset).

Next, we tested if the fish could still discriminate when the difference between targets was reduced to only one food pellet. Thus, with the same setup we examined whether the fish could distinguish between three and four (Fig 4C), after an extinction phase. Here, we observed a variety of learning capabilities in the fish population. One fish clearly learned to choose the most highly rewarded target (Fish 4, Fig 4C), whereas another fish did not change its preference, with an initial and final rate of about 40% (Fish 8, Fig 4C). Another three fish changed their preference from the original to about an equal proportion for both targets. Although the final success rate (53%) in this experiment was lower than in the previous ones with a difference of two food pellets, it was still significantly higher than the initial rate (15%, p<0.0001).

#### Three shape-value association

**Fig 5:**
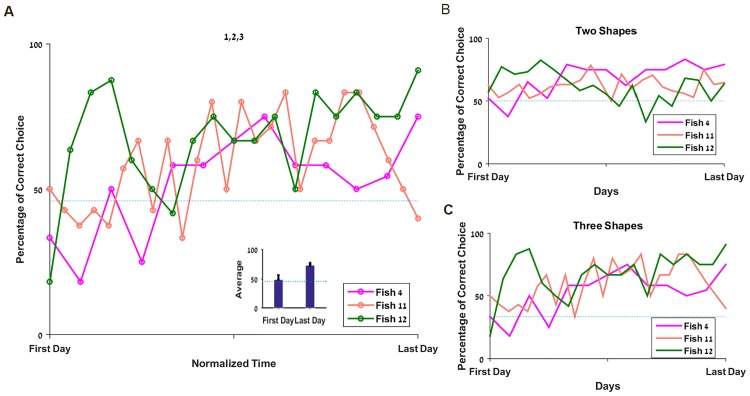
Fish can associate three values and with three shapes. **A**. Success rate for each fish on the 1, 2, 3 experiment over normalized time. Inset: average success rate on the first and last day. *The dashed line represents the chance level (45*.*83%)*. **B**. Success rate for the three combinations with two targets. *The dashed line represents the chance level (50%)*. **C.** Success rate for the combination with three targets presented simultaneously. *The dashed line represents the chance level (33%)*.

To test the ability of the fish to associate values with symbols in a more complex setup, we tested the fish with three shapes associated with three amounts of reward. Spitting at the triangle was associated with one food pellet, two when the square was chosen and three for the circle. The results showed that although the initial success rate was close to chance, the fish eventually learned to choose the most rewarded targets with an average rate of about 70% (p<0.0001, Fig 5A).

When breaking down the results between the three pair comparisons (1 versus 2, 1 versus 3 and 2 versus 3, Fig 5B) and the one with three shapes (Fig 5C), it appears clearly that Fish 4 was able to choose the most rewarded target in all conditions. Fish 11 and 12 succeeded significantly above chance with three targets but their overall results for the two shapes combination do not allow us to clearly state that they succeeded.

**Fig 6:**
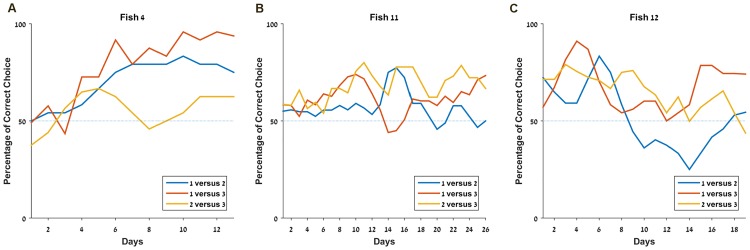
Some fish are able to organize shapes according to the value associated with them. **A.** Details of the experiment with three shapes for the pairs comparisons for Fish 4. **B.** Details of the experiment with three shapes for the pairs comparisons for fish 11. **C.** Details of the experiment with three shapes for the pairs comparisons for fish 12.

Since the fish encountered each pair of shapes only nine times per session and may not have spitted every time, the results’ variance is high. Thus, to increase the significance of the observations, we chose to analyze in Fig 6 the moving average (3 days) of the success rate. This more detailed depiction of results for the pair conditions reveals that only Fish 4 (Fig 6A) shows an upward trend for each combinations, whereas the two other fish seem to oscillate (Fig 6B and 6C). It is also important to notice that since we conducted the experiment until we saw a steady state in the overall score rather than in each subcategory separately, we cannot state that the fish were unable to pick the correct answer in the three two-shape combinations. On the contrary, the fact that Fish 4 succeeded suggests that given enough time, the others might also be able to reach a stable success rate above chance level in every condition.

## Discussion & conclusion

Previous studies revealed that animals such as primates or birds are able to understand and manipulate symbolic representation of quantities [21–27]. Here we show for the first time that fish are also able of such an abstraction.

Building on the natural preference for shapes in archerfish provided a powerful tool to investigate decision making, and made shifts in preference more salient. Almost all the fish became proficient in the tasks and successfully shifted away from their initial preference toward the target associated with the largest food reward. This suggests that fish are able to associate different abstract symbols with different quantities of reward. The different experiments with two targets revealed that the fish are not only able to discriminate between ‘one’ and ‘many’ but also between two quantities bigger than one, and do so even when the difference between the two is smaller.

While the final choice rates in the third experiment (3 versus 4) were roughly equal, this result nevertheless indicates that fish perceived a difference between the two values. Indeed, had they estimated the rewards as equal, they would have kept choosing the triangle just like they did during the natural preference phase, when all targets led to the same reward (Fig 2). Thus this experiment indicates that the fish can discriminate between quantities that are separated by only one unit, at least when the rewards are smaller or equal to four.

To determine whether the fish were able to associate more than two values with symbols, we conducted the three shapes experiment. The difficulty of the decision in this experiment was due to the fact that the combination of symbols changed every trial. Thus, the fish needed an organized representation of the values associated with each shape in order to be able to mentally compare them to each other in every set of choice. Although the progress from the initial to final success rates was considerably lower than in the previous experiments, it remained significantly higher than chance indicating that fish can compare more than two values and organize them mentally.

### Success rates depend on ratio of rewards

Current theories about the existence of an organized representation of magnitudes in humans and animals [35,36] are based on the existence of a correlation between the success rate when comparing two values and the ratio between those two. Studies have shown in humans and other primates that accuracy decreases and latency increases as the ratio between two values gets larger [35,37] and it is true even during comparison [36,38] of symbolic representations.

**Fig 7:**
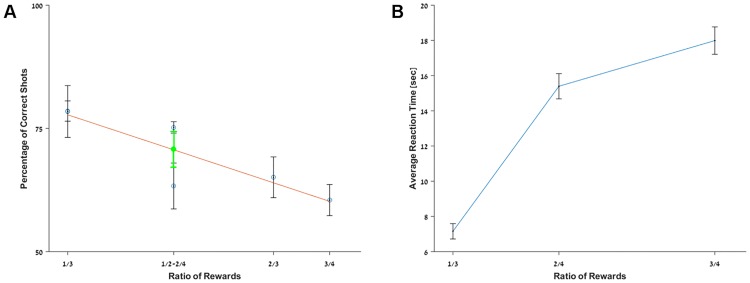
Ratio effect and fish reaction. **A**. Effect of value ratio over success rate; the values represent the average over the last three days of the experiment. The error bars represent the standard error. The linear regression is depicted in red. **B**. Effect of value ratio over reaction time.

Fig 7A shows the average success rate over the last three days of each experiment of two-shape comparison. We also show the results of the fourth experiment (with three shapes) for the three conditions with two shapes. Scores for the two experiments of 1 versus 3 (first and fourth experiment) are identical and their representations on the graph are not distinguishable. The other scores also follow closely the linear regression. The only outlier is the result for 1 versus 2 in the three-shape experiment that falls below the general trend. However, the mean score for the two experiments with the 0.5 ratio (green dot) falls exactly on the predictive curve.

Interestingly, the final success rates per pair for Fish 4 (Fig 6A) also follow exactly this prediction and Fish 12 (Fig 6C) seems to be on the same track. The analysis of the success rates in pair choices thus suggests the existence of a ratio effect in the archerfish as predicted by Weber’s law [39], hinting the existence of an organized system to represent magnitudes.

To confirm this intuition we also analyzed reaction times. If the fish made a binary decision (‘less’ vs. ‘more’), the reaction time should not change as the distance between the associated rewards varies. By contrast, if fish are able to map the values associated with each target on a mental magnitude line [9], reaction time should rise as the comparison becomes harder, as observed in humans and other animals [40,41].

Fig 7B shows the average reaction times across all fish during the last day of the experiment as a function of the ratio of rewards. We did not include the last experiment with the three shapes in this analysis since the number of choices increased the complexity of the experiment exponentially and thus impacted the reaction time of the fish. This analysis reveals that as the ratio of reward approaches one, the reaction time increases, indicating that the task became harder for the fish and confirming the existence of an organized mental representation of values. The results are also coherent with symbol discrimination in humans [42], where the time to determine which one of two Arabic numbers is the biggest also depends on their ratio.

### Continuous magnitudes as representation of quantities

It was thought for a long time that humans and some animals possess a discrete representation of quantities [9,36]. However, recent studies suggest that it is virtually impossible to remove all influence of continuous magnitudes [43], and thus it seems more likely that animals and humans developed instead a continuous representation of magnitudes. We try here to identify the potential magnitudes used by the fish to determine the value of rewards associated with a target. Yet, although it appears clearly that the archerfish successfully did so, our experiments did not permit to determine which parameters the fish used to encode those values.

Our behavioral experiments were not based on a simple visual comparison task as was conducted with other fish [10–13,15]. Indeed, the two values to be compared, the number of food pellets, are never present simultaneously. Therefore, the fish need to encode and store the value they attribute to each symbol in order to mentally compare it with the values they will encounter later. Our experiments show some similarities with the one conducted by Dadda [17] where mosquitofish saw members of a group sequentially and not all at once. However, since this setup did not use symbols, the mosquitofish could easily base their decision on some continuous magnitude directly linked to the stimulus such as the time it takes to see all the individuals.

It seems very unlikely that the archerfish based their choice on only one continuous magnitude linked to the amount of food pellets. Indeed, the food repartition was influenced by too many factors. Since the experimenter always dropped the food on the same side of the aquarium, the fish rapidly learned to position themselves where the pellets would fall, just as they do when they hunt [44]. Thus, they often caught some food when it reached the water or even right before, while the other pieces started floating and spreading randomly immediately because of the flow generated by the air pump. In addition, the fish are close to the surface when they expect food, giving them a maximum viewing angle of 90° [45] that does not encompass the whole aquarium’s surface at once, potentially preventing them from seeing all the food. Therefore, it is likely that if the fish used visual continuous magnitudes, they needed to use all available variables (density, covered area, etc.) and not only one to evaluate the amount of food present.

It is also possible that the fish uses additional magnitudes to determine which symbol yields more food. For example, satiety could be a parameter, or the effort required to eat all the food pellets (time, physical effort…). However, the random dispersion of the food pellets renders those magnitude very noisy, which is coherent both with the observed ratio effect observed and the literature [36].

Although many factors can influence the continuous magnitudes, since we do not have a way to equate all of them (time to grab, dispersion, satiety …) and they are correlated with the number of food pellets, it is likely that the fish used those magnitudes as cues to determine how much food they got. We can thus only conclude that the fish possess a magnitude sense [46,47] of the amount of reward. The influence of continuous magnitudes is not crucial for the conclusions of the current work since it is possible that the symbols are connected with both number and continuous magnitudes, as demonstrated in humans [48]].

### Effect of learning on success rate

It is worthwhile noticing that not all the fish showed a similar level of success in the different tasks. Several factors could explain this effect: it is probable that just like in humans [49], there is a range of cognitive abilities and not all the fish present the same aptitudes. A recent review of cognitive experiments performed with fish also suggests the existence of such a phenomenon [50]. Additionally, they did not all perform the whole set of experiments. Only Fish 4 went through each step and it is interesting to see that it is also the fish with the best results in the two last experiments. This could indicate that the ability to associate a value with a shape can be trained and improved and that with enough practice, archerfish could succeed in tasks more complex than the ones tested here.

To conclude, our experiments revealed that archerfish are able to associate values of rewards with abstract symbol, compare them and consistently choose the more rewarding target. We also saw that their success rate and reaction time are correlated with the ratio between the rewards. These results suggest that archerfish possess an organized mental representation of magnitudes that enables them to perform comparisons, rather than a simple binary notion of ‘more’ and ‘less’.

## Supporting information

S1 Data Experimental raw data. One line correspond to a session for tabs 1–6 and first part of tab 7. Second part of tab 7 contains the details of each session. Tab 8 contains the number of shot during the last session as well as the reaction times.
(XLSX)
